# Impact of Hypogammaglobulinemia on the Course of COVID-19 in a Non-Intensive Care Setting: A Single-Center Retrospective Cohort Study

**DOI:** 10.3389/fimmu.2022.842643

**Published:** 2022-03-10

**Authors:** Riccardo Scarpa, Alessandro Dell’Edera, Carla Felice, Roberta Buso, Francesco Muscianisi, Renato Finco Gambier, Sara Toffolo, Ugo Grossi, Mario Giobbia, Giuseppina Barberio, Nicholas Landini, Cesarina Facchini, Carlo Agostini, Marcello Rattazzi, Francesco Cinetto

**Affiliations:** ^1^ Internal Medicine I, Ca’ Foncello Hospital, Azienda Unità Locale Socio Sanitaria n. 2 (AULSS2) Marca Trevigiana, Treviso, Italy; ^2^ Department of Medicine, University of Padova, Padua, Italy; ^3^ Department of Surgery, Ca’ Foncello Hospital, Azienda Unità Locale Socio Sanitaria n. 2 (AULSS2) Marca Trevigiana, Treviso, Italy; ^4^ Infectious Diseases Unit, Ca’ Foncello Hospital, Azienda Unità Locale Socio Sanitaria n. 2 (AULSS2) Marca Trevigiana, Treviso, Italy; ^5^ Laboratory Medicine, Ca’ Foncello Hospital, Azienda Unità Locale Socio Sanitaria n. 2 (AULSS2) Marca Trevigiana, Treviso, Italy; ^6^ Radiology Unit, Ca’ Foncello Hospital, Azienda Unità Locale Socio Sanitaria n. 2 (AULSS2) Marca Trevigiana, Treviso, Italy

**Keywords:** Hypogammaglobulimenia, COVID-19, internal medicine (INT), antibody deficiency, immunodeficiency, immunoglobulin replacement therapy (IGRT), severity, SARS-CoV-2

## Abstract

**Background:**

Severity and mortality of COVID-19 largely depends on the ability of the immune system to clear the virus. Among various comorbidities potentially impacting on this process, the weight and the consequences of an antibody deficiency have not yet been clarified.

**Methods:**

We used serum protein electrophoresis to screen for hypogammaglobulinemia in a cohort of consecutive adult patients with COVID-19 pneumonia, hospitalized in non-intensive care setting between December 2020 and January 2021. The disease severity, measured by a validated score and by the need for semi intensive (sICU) or intensive care unit (ICU) admission, and the 30-day mortality was compared between patients presenting hypogammaglobulinemia (HYPO) and without hypogammaglobulinemia (no-HYPO). Demographics, comorbidities, COVID-19 specific treatment during the hospital stay, disease duration, complications and laboratory parameters were also evaluated in both groups.

**Results:**

We enrolled 374 patients, of which 39 represented the HYPO cohort (10.4%). In 10/39 the condition was previously neglected, while in the other 29/39 hematologic malignancies were common (61.5%); 2/39 were on regular immunoglobulin replacement therapy (IgRT). Patients belonging to the HYPO group more frequently developed a severe COVID-19 and more often required sICU/ICU admission than no-HYPO patients. IgRT were administered in 8/39 during hospitalization; none of them died or needed sICU/ICU. Among HYPO cohort, we observed a significantly higher prevalence of neoplastic affections, of active oncologic treatment and bronchiectasis, together with higher prevalence of viral and bacterial superinfections, mechanical ventilation, convalescent plasma and SARS-CoV-2 monoclonal antibodies administration during hospital stay, and longer disease duration. Multivariate logistic regression analysis and Cox proportional hazard regression confirmed the impact of hypogammaglobulinemia on the COVID-19 severity and the probability of sICU/ICU admission. The analysis of the mortality rate in the whole cohort showed no significant difference between HYPO and no-HYPO.

**Conclusions:**

Hypogammaglobulinemia, regardless of its cause, in COVID-19 patients hospitalized in a non-intensive care setting was associated to a more severe disease course and more frequent admission to s-ICU/ICU, particularly in absence of IgRT. Our findings emphasize the add-value of routine serum protein electrophoresis evaluation in patients admitted with COVID-19 to support clinicians in patient care and to consider IgRT initiation during hospitalization.

## Introduction

Since the beginning of the SARS-CoV2 pandemic in 2019, clinical and experimental evidence has tried to dissect mechanisms of disease and to identify the critical elements to improve the management of patients. To date, the picture is far to be achieved, but the presence of pre-existing comorbidities has been recognized as a risk factor associated with disease severity (1) and the prognostic role of chronic lung and heart disease, obesity, diabetes, and arterial hypertension has been highlighted (2, 3). Particularly, patients admitted to semi- (sICU) and intensive care unit (ICU) due to SARS-CoV-2 infection had, on average, more comorbidities than those hospitalized in non-ICU (4, 5). On the contrary, the consequences of SARS-CoV-2 infection in individuals with primary and secondary antibody deficiencies are still unclear (6, 7). In one of the first published reports of 7 Italian cases of COVID-19 in patients affected by primary antibody deficiency (PAD) described a more severe clinical course of COVID-19 in common variable immunodeficiency than in X-linked agammaglobulinemia patients, suggesting that a B cell dysfunction may be more dangerous than the complete lack of antibody production (8). It is indeed broadly recognized that a profound interplay exists between the host immune response and the COVID-19 course both in terms of susceptibility and clinical outcome (9). As a result, different published cohorts of patients with inborn errors of immunity (IEIs) reported mixed results depending on the underlying immunological defect (10). A multicenter retrospective study investigated the impact of SARS-CoV-2 infection on 94 patients with a spectrum of IEIs, mainly antibody deficiencies, reporting that disease severity and mortality were globally similar to those in the general population (11). When evaluating these findings, it is important to consider that almost all patients with PAD enrolled in those studies were receiving immunoglobulin substitution as standard therapy, thus presenting adequate serum IgG trough levels at the time of SARS-CoV-2 infection. On the contrary, the observations derived from hematologic patients, when a severe hypogammaglobulinemia was detected without mention of immunoglobulin replacement therapy (IgRT), COVID-19 was associated with higher mortality and severe inflammatory response compared to the general population (12, 13).

Apart from scan data coming from hematological perspective, the specific impact of pre-existing antibody deficiency on COVID-19 has not been yet investigated. Antibody deficiencies encompass a group of several disorders resulting in hypogammaglobulinemia, that can be can be distinguished into primary (PAD) and secondary (SAD) to specific diseases/treatments, leading to an increased susceptibility to a severe course of infections and abnormal antibody response to immunizations (11, 14). According to the origin of the impaired differentiation of B cells into immunoglobulin-secreting plasma cells they may also bear higher risk of dysregulated inflammatory responses (15).

Thus, we decided to investigate the prevalence of hypogammaglobulinemia in a cohort of patients with microbiological diagnosis of SARS-CoV-2 infection admitted to an Internal Medicine Unit, in order to point out its impact on COVID-19 severity and mortality.

## Materials and Methods

### Study Design and Objectives

We conducted a single-center retrospective cohort study in which we enrolled consecutive adult patients with COVID-19 pneumonia and microbiological diagnosis of SARS-CoV-2 infection, admitted between the December 1, 2020 and January 31, 2021 to the Internal Medicine Unit of Ca’ Foncello University Hospital (Department of Medicine, University of Padova, AULSS2, Treviso, Italy). Each diagnosis of SARS-CoV-2 infection was made using semi-quantitative real-time reverse transcription-polymerase chain reaction (RT-PCR) on a nasopharyngeal swab, according to international guidelines. Patients were admitted to medical wards directly from the Emergency Department and according to the local protocols. Inclusion criteria were a routine serum protein electrophoresis (SPEP) available at admission and informed consent to the study.

The study was approved by the local institutional review board and was performed in accordance with the Declaration of Helsinki. All participants were enrolled in the AULSS2 COVID-19 Registry (793/CE Marca Trevigiana). Data were obtained from medical records and entered into an anonymous database, namely, demographics, comorbidities, disease specific treatments, complications, disease timeline, and laboratory parameters. Need and type of ventilation support and need for sICU/ICU admission and cause of death were also recorded.

### Definitions

On the basis of the routine SPEP performed at the admission, hypogammaglobulinemia was defined as a gamma-globulin fraction below 9%, subsequently confirmed by the evidence of a serum IgG level below 600 milligrams per deciliter (mg/dl). We set thresholds lower than those commonly used by our laboratory (gamma-globulin <10%, IgG <700 mg/dl) for the inclusion in the hypogammaglobulinemia cohort, in order to exclude borderline and transient hypogammaglobulinemia.

A previously validated, COVID-19 specific seven-category ordinal scale was used to estimate disease severity in terms of degree of ventilation needed, and the highest level achieved during the hospitalization was considered (16). We defined as severe a score of 5 points or higher, corresponding to the “severe” category of the WHO clinical progression scale (17). The seven-category ordinal scale consisted of the following categories: 1, not hospitalized with resumption of normal activities; 2, not hospitalized, but unable to resume normal activities; 3, hospitalized, not requiring supplemental oxygen; 4, hospitalized, requiring supplemental oxygen; 5, hospitalized, requiring nasal high-flow oxygen therapy, noninvasive mechanical ventilation, or both; 6, hospitalized, requiring ECMO, invasive mechanical ventilation, or both; and 7, death.

Criteria for semi-intensive care unit (s-ICU) or ICU admission were established by local protocols and remained unchanged throughout the observation period, namely, P/F ratio <200, respiratory rate >30 breaths/min, septic shock with need for vasopressors, respiratory failure requiring mechanical ventilation. Finally, time to event (mortality, sICU/ICU admission) was measured since hospital admission.

### Primary and Secondary Endpoints

The primary endpoints of our study were the prevalence of hypogammaglobulinemia in a cohort of COVID-19 patients admitted to a non-intensive care unit and its impact on all-cause mortality within 30 days from the hospital admission and on disease severity, measured as need for s-ICU or ICU admission and as a seven-category score ≥5.

Secondary endpoints of our study included:

- estimation of the impact of hypogammaglobulinemia on time to hospitalization (time from the microbiological diagnosis of SARS-CoV-2 infection to the hospital admission), disease duration (time to viral clearance from the microbiological diagnosis of SARS-CoV-2 infection to the second negative RT-PCR swab over a period of 24 h) and length of hospital stay (time from hospital admission to discharge);
- describing, in the two cohorts of patients with hypogammaglobulinemia (HYPO) and without hypogammaglobulinemia (no-HYPO), demographics (age, sex), co-morbidities known as possible predictors of COVID-19 severity (arterial hypertension, dyslipidemia, diabetes mellitus, ischemic heart disease, obesity defined as BMI ≥30, bronchiectasis, history of cancer, active cancer treatment), diseases specific treatment during the hospital stay (steroids, Remdesivir, anti-SARS-COV-2 monoclonal antibodies, convalescent plasma, polyclonal immunoglobulins, anticoagulation, antibiotics), disease-related complications (superinfections, pulmonary thromboembolism) and also laboratory parameters, namely, C-reactive protein (CRP), total white blood cell (WBCs) and differential count, procalcitonin (PCT), IL-6 (interleukin-6), ferritin, Erythrocyte Sedimentation Rate (ERS) and D-Dimer;
- comparing the above-mentioned parameters between hypogammaglobulinemic patients admitted (HYPO-ICU) or not (HYPO no-ICU) to the semi-intensive/intensive care unit.

### Statistical Analysis

We used Mann–Whitney test to compare quantitative variables across two groups, respectively. We reported median and interquartile range (IQR) as descriptive statistics for continuous variables. Chi-squared and Fisher’s exact tests were used for categorical variables. Binomial logistic regression models were fitted to calculate odds ratios (OR) with 95% confidence intervals (CI) for the need of s-ICU/ICU admission. Multivariable logistic regression analysis was then performed, to confirm the findings for primary endpoints, taking into consideration appropriate covariates selected from anamnestic clinical and laboratory parameters. Kaplan–Meier survival analysis was also performed to investigate the 30-day mortality and the sICU/ICU admission free survival between HYPO and no-HYPO groups. The log rank (Mantel–Cox) test was used for the comparison between the survival curves of the two groups. The Cox proportional hazard regression was then used to adjust survival analysis for selected confounders. Statistical significance was considered as a two-tailed p <0.05. All the analyses were performed using IBM SPSS statistics 27.0.

## Results

### Study Population

**Figure 1:**
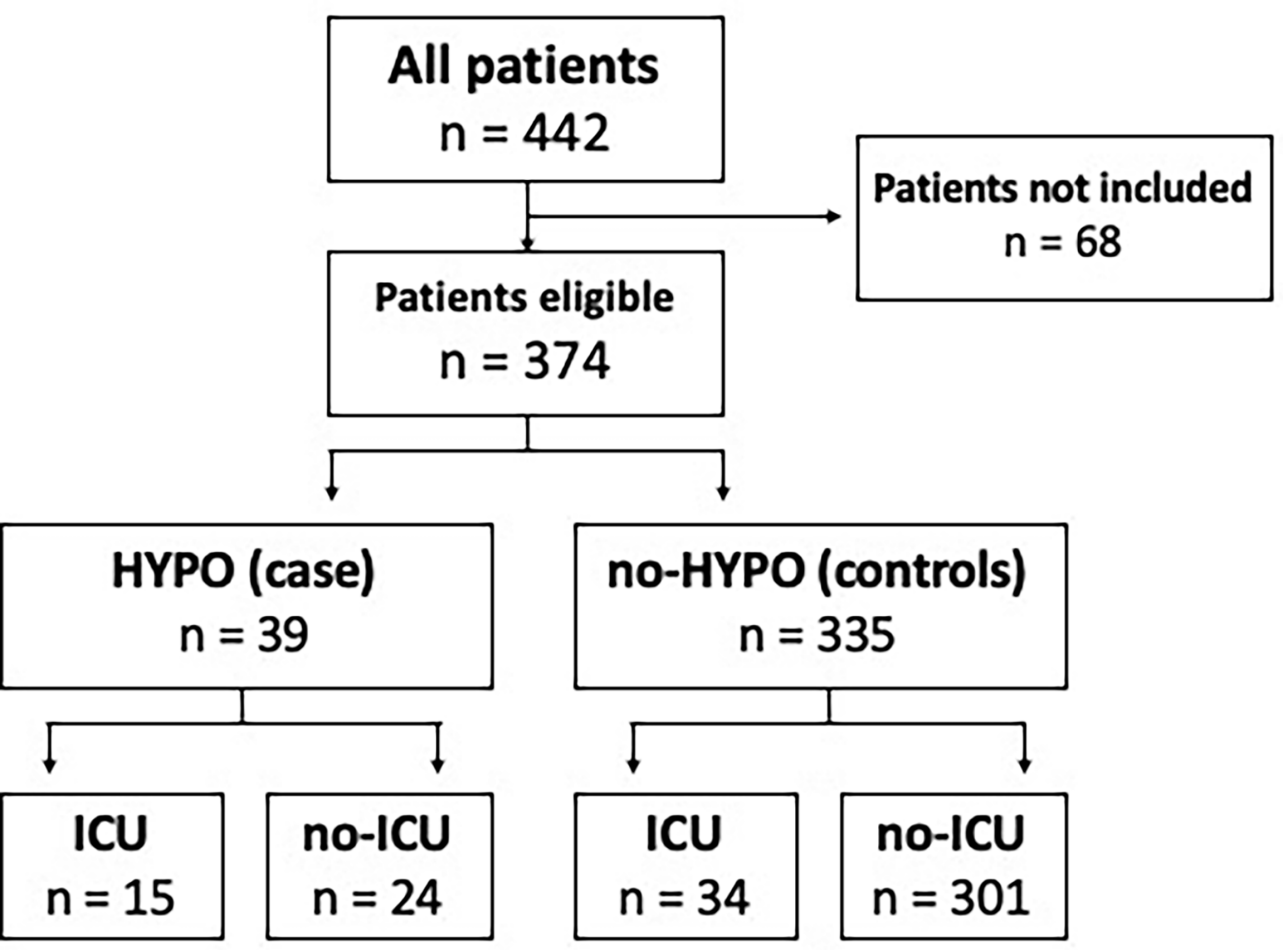
Distribution of hypogammaglobulinemia and ICU admission in our COVID-19 cohort. A total of 68 patients were not included due to lack of serum protein electrophoresis, informed consent and/or not eligible for s-ICU/ICU. HYPO, hypogammaglobulinemia; ICU, intensive care unit.

From December 1, 2020 to January 31, 2021, 442 consecutive patients were admitted to our Internal Medicine Unit with a diagnosis of COVID-19 pneumonia. Based on the inclusion criteria, we enrolled 374 patients (84.6% of the whole population). We found 39 out of 374 patients presenting hypogammaglobulinemia (HYPO, cases) and 335 without this condition (no-HYPO, controls). Prevalence of hypogammaglobulinemia was 10.4% (**Figure 1**).

A total of 10 out of 39 patients with hypogammaglobulinemia (25.5%) received a new diagnosis of hypogammaglobulinemia of unknown etiology during the hospitalization (**Supplementary Figure 1**), despite low gamma globulin count having been already detected in the clinical records of all subjects before the admission. Among the other 29 of 39 patients with defined etiology of hypogammaglobulinemia, we found that hematological malignancies were the most common cause (61.5%, n = 24), followed by solid tumors (5%, n = 2) and, lastly, by two cases of rheumatological affections (vasculitis and glomerulonephritis) and only one case of primary antibody deficiency. Taking into consideration the hematological subgroup, **Supplementary Figure 2** shows a higher prevalence of monoclonal gammopathy, both of unknown significance (MGUS, 21% n = 5) and in the context of multiple myeloma (MM, 25% n = 6), rather than Non-Hodgkin Lymphomas (NHL, 21% n = 5) and Chronic Lymphocytic Leukemia (CLL, 8.5% n = 2). We found only few cases of other hematological disorders, like acute myeloid leukemia (n = 2), Hodgkin lymphoma (n = 1), T-cell lymphoma (n = 1), myelodysplastic syndrome (n = 1) and one amyloidosis (n = 1).

### HYPO Versus No-HYPO Cohort

The baseline characteristics of the 374 patients included in the study are summarized in **Table 1**. In terms of demographics, the median age was similar in both HYPO and no-HYPO groups (72 vs. 74 years, *p* = 0.611) and also the gender distribution (M 46% vs 63%, p = 0.068). Among HYPO cohort, we observed a significantly higher prevalence of neoplastic affections and of active oncologic treatment (66.7% vs. 20.9% for cancer and 47.4% vs. 2.4% for active treatment, *p <*0.001), together with a higher prevalence of bronchiectasis (23.1% vs. 1.2%, *p <*0.001) and anamnestic corticosteroid usage (41% vs. 9.6%, *p <*0.001) as compared to the no-HYPO cohort. On the contrary, no differences were found in cardiovascular and metabolic comorbidities like arterial hypertension, dyslipidemia, diabetes mellitus, ischemic heart disease and obesity.

**Table 1:** Demographics and comorbidities between HYPO vs. no-HYPO in our COVID-19 cohort.

	*HYPO(n = 39)*	*no-HYPO(n = 335)*	p*
**Age—**years (IQR)	72 (62–82)	74 (63–83)	0.611
**Gender—**% Male	46%	63%	0.068
**Comorbidities** n (%)			
*Cancer*	26 (66.7%)	70 (20.9%)	**<0.001**
*Hematologic malignancies*	24 (62%)	8 (2.4%)	**<0.001**
*Active cancer treatment*	18 (47.4%)	8 (2.4%)	**<0.001**
*Bronchiectasis*	9 (23.1%)	4 (1.2%)	**<0.001**
*Previous corticosteroid therapy*	16 (41%)	32 (9.6%)	**<0.001**
*Arterial hypertension*	19 (49%)	215 (64%)	0.087
*Dyslipidemia*	12 (31%)	62 (19%)	0.350
*Diabetes mellitus*	6 (15%)	75 (22%)	0.350
*Ischemic heart disease*	7 (18%)	36 (11%)	0.160
*Obesity*	5 (13%)	55 (16%)	0.652

Categorical variables were reported as percentages of the total. The sex ratio is expressed as male percentage of the total. *Chi-squared test. HYPO, hypogammaglobulinemia.

Bold font highlights statistically significant p values.

Considering the therapeutic strategies during the hospital stay, the use of systemic corticosteroid therapy showed a higher prevalence in the no-HYPO group (72% vs. 96%, *p <*0.001, **Table 2**). We found no difference in the use of remdesivir (27% vs. 43%, *p* = 0.080), anticoagulant prophylaxis (92.3% vs. 92.2%, *p* = 0.999) and anticoagulant therapy (7.7% vs. 7.8%, *p* = 0.999). On the contrary, the HYPO group showed higher prevalence of non-invasive and/or invasive mechanical ventilation (66.7% vs. 22.1%, *p <*0.001), convalescent plasma (12.8% vs. 0.6%, *p <*0.001) and SARS-CoV-2 monoclonal antibodies (5.3% vs. 0%, *p <*0.05). Polyclonal immunoglobulins were administered as replacement therapy only in 8 patients of the HYPO group (20.5% vs. 0%, *p <*0.001). Finally, a significant difference was detected in antibiotic usage (64% HYPO vs. 9% no-HYPO, *p <*0.001).

**Table 2:** Treatments, laboratory parameters, clinical complications and disease duration between HYPO vs. no-HYPO in our COVID-19 cohort.

	*HYPO(n = 39)*	*no-HYPO(n = 335)*	p*
**Treatments—**n (%)			
*Steroid therapy*	28 (72%)	324 (96%)	**<0.001**
*Remdesivir*	10 (27%)	167 (43%)	0.080
*Anticoagulant prophylaxis*	36 (92.3%)	309 (92.2%)	0.999
*Anticoagulant therapy*	3 (7.7%)	26 (7.8%)	0.999
*Mechanical ventilation^1^*	26 (66.7%)	74 (22.1%)	**<0.001**
*Convalescent plasma*	5 (12.8%)	2 (0.6%)	**<0.001**
*SARS-CoV-2 Monoclonal Antibodies*	2 (5.1%)	0 (0%)	**0.010**
*Polyclonal Immunoglobulins*	8 (20.5%)	0 (0%)	**<0.001**
*Antibiotics*	25 (64%)	30 (9%)	**<0.001**
**Laboratory—**unit (IQR)			
*WBCs* (mm^3^)	8.3 (6.7–10)	6.58 (4.6–8.9)	**0.006**
*Lymphocytes* (mm^3^)	0.9 (0.56–1.25)	0.9 (0.61–1.18)	0.837
*CRP* (mg/dl)	13.7 (4.9–19.4)	6.39 (2.9–12.4)	**0.005**
*ERS* (mm/h)	67 (40–80)	46 (29–65)	0.207
*PCT* (ng/ml)	0.59 (0.05–2.25)	0.11 (0.06–0.31)	0.573
*IL-6* (mg/dl)	16.9 (11.5–35.5)	16.5 (10–67.9)	0.856
*D-Dimer* (ng/l)	1,589 (971–8,191)	934 (589–1,560)	0.862
*Ferritin* (ng/ml)	532 (332–2,510)	691 (394–1,109)	0.711
**Complications and disease duration**			
*Superinfections*	19 (48.7%)	27 (8.1%)	**<0.001**
*Thromboembolism*	1 (2.6%)	19 (5.7%)	0.708
*Disease duration—days (IQR)*	31 (20–40)	19 (14–23)	**<0.001**
*Time to hospitalisation—days (IQR)*	7 (4–9)	6 (3–9)	0.452
*Length of hospital stay—days (IQR)*	17 (13–24)	6 (4–9)	**<0.001**

Quantitative variables are expressed as median and IQR. Categorical variables were reported as percentages of the total. *Chi-squared and Mann–Whitney test. ^1^Mechanical ventilation includes non-invasive and invasive.

HYPO, hypogammaglobulinemia; WBCs, total white blood cell; CRP, C-reactive protein; ERS, erythrocyte sedimentation rate; PCT, procalcitonin; IL-6, interleukin-6.

Bold font highlights statistically significant p values.

When analyzing the laboratory parameters, we found a significant difference in CRP levels between cases and controls (13.7 and 6.39 mg/dl, respectively, *p <*0.05, **Table 2**). The HYPO cohort also presented a significant increase in WBC count (8.3 vs. 6.58/mm^3^, *p* = 0.006) without difference in lymphocytes count as compared to controls. No differences were found for ESR, PCT, D-Dimer, IL-6, and ferritin.

Focusing on clinical complications, we found a significantly higher prevalence of viral and bacterial superinfections in the HYPO group (48.7% vs. 8.1%, *p <*0.001, **Table 2**), especially considering *Staphylococcus aureus*, Pneumococcal *pneumonia* and opportunistic infections (e.g., *Cytomegalovirus* and *Aspergillus fumigatus*). **Supplementary Table 1** reports the different pathogens isolated in the microbiological samples (blood cultures, urine cultures and swabs). No difference was found in terms of thromboembolism events between the two groups during the hospital stay (2.6% vs. 5.7%, *p* = 0.708, **Table 2**).

As summarized in **Table 2**, when considering the temporal course of SARS-CoV-2 infection, the HYPO group showed a significantly longer disease duration (31 vs. 19 days, *p <*0.001) and length of hospital stay (17 vs. 6 days, *p <*0.001), but no difference was found in time to hospitalization (7 vs. 6 days, *p* = 0.452).

### COVID-19 Severity and Mortality

Considering the maximum degree of the seven-category scale achieved during the hospitalization, we registered an increased prevalence of patients requiring non-invasive and invasive mechanical ventilation (Score ≥5) in the HYPO group (27 vs. 74 patients, *p <*0.001, **Table 3**). On the contrary, we found a significant prevalence of subjects requiring oxygen support with a low-flow system, by nasal-cannula or simple face mask (Score = 4) in the no-HYPO group (12 vs. 255 patients, *p <*0.001, **Table 3**). Binomial logistic regression analysis showed a positive correlation between disease severity (Score ≥5) and hypogammaglobulinemia (OR 7.94 95% CI 3.83–16.42, *p <*0.001, **Table 4**).

**Table 3:** The seven-category scale represents the maximum clinical status of patients achieved during the COVID-19 hospitalization.

Seven-category Scale	HYPO (n = 39)	no-HYPO (n = 335)	p*
3: hospitalization, not requiring supplemental oxygen—no. (%)	0 (0%)	6 (1.8%)	0.999
4: hospitalization, requiring supplemental oxygen—no. (%)	12 (30.8%)	255 (76.1%)	**<0.001**
≥5: hospitalization, requiring mechanical ventilation—no. (%)	27 (69.2%)	74 (22.1%)	**<0.001**
*5: hospitalization, requiring HFNC or non-invasive mechanical ventilation—no. (%)*	16 (41%)	21 (6.3%)	**<0.001**
*6: hospitalization, requiring ECMO, invasive mechanical ventilation, or both—no. (%)*	2 (5.1%)	0 (0%)	**0.010**
*7: death—no. (%)*	9 (23.1%)	53 (15.8%)	0.256

*Chi-squared test.

HYPO, hypogammaglobulinemia; HFNC, High Flow Nasal Cannula; ECMO, ExtraCorporeal Membrane Oxygenation.

Bold font highlights statistically significant p values.

**Table 4:** Primary outcomes in the overall population.

Disease severity (score ≥5)		Unadjusted		Adjusted	
	**n (%)**	**OR (95% CI)**	p*	**OR (95% CI)**	**p^§^**
HYPO vs. no-HYPO	27 (69.2%) vs. 74 (22.1%)	7.94 (3.83-16.42)	**<0.001**	8.52 (3.31-21.93)	**<0.001**
**s-ICU/ICU-Admission**		**Unadjusted**		**Adjusted**	
	**n (%)**	**OR (95% CI)**	p*	**OR (95% CI)**	**p^§^**
HYPO vs. no-HYPO	15 (38.5%) vs. 34 (10%)	5.53 (2.65-11.55)	**<0.001**	5.35 (2.08-13.78)	**<0.001**
**30-Day Mortality**		**Unadjusted**		**Adjusted**	
	**n (%)**	**OR (95% CI)**	p*	**OR (95% CI)**	**p^§^**
HYPO vs. no-HYPO	9 (23%) vs. 53 (16%)	1.60 (0.72-3.56)	0.252	1.88 (0.66-5.36)	0.240

^*^Binomial logistic regression analysis.

^§^Adjusted for age, gender, presence of neoplasia, steroid treatment by multivariable logistic regression analysis.

HYPO, hypogammaglobulinemia; ICU, intensive care unit; s-ICU, semi-intensive care unit.

**Figure 2:**
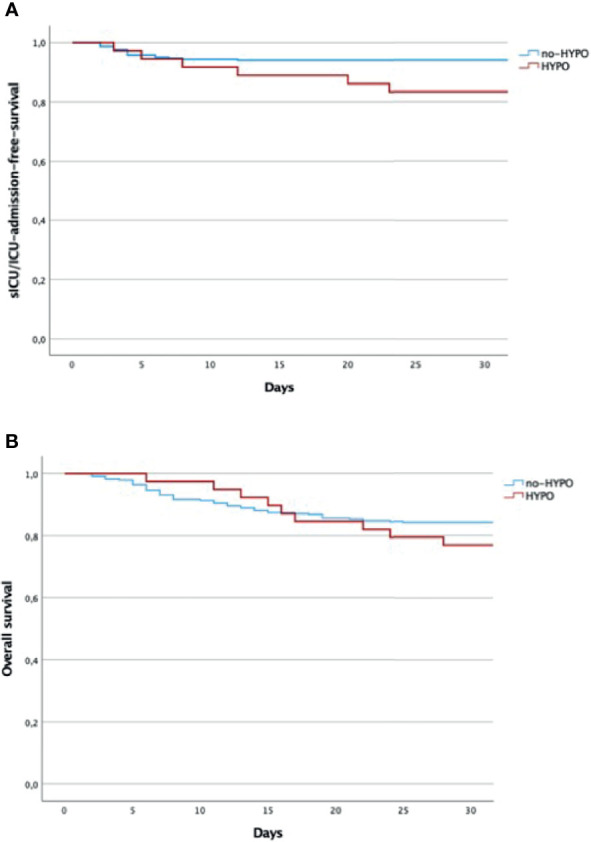
**(A)** Overall 30-days mortality (days from the admission to the hospital). Log rank test: χ^2^(1) = 0.992, *p* = 0.319. **(B)** s-ICU/ICU-admission-free survival (days from the admission to the hospital). Log rank test: χ^2^(2) = 23.62, *p <* 0.001. Kaplan–Meier analysis by the log-rank test. HYPO, hypogammaglobulinemia; ICU, intensive care unit; s-ICU, semi-intensive care unit.

Among the whole population, we also found a subgroup of 49 subjects requiring sICU/ICU admission, of which 15 HYPO and 34 no-HYPO. Patients belonging to the HYPO group were more frequently admitted to sICU/ICU than patients without hypogammaglobulinemia (38.5% vs. 10%, OR 5.53 95% CI 2.65–11.55, *p <*0.001, **Table 4**). The analysis of the mortality rate in the whole cohort showed no significant difference between cases and controls (23% vs. 16%, OR 1.60, *p* = 0.394, **Table 4**), with an overall 30-day mortality (16,5%) in line with that reported in the same region. The Kaplan–Meier survival analysis confirmed these findings, showing an overlap in the curves of HYPO vs. no-HYPO for 30-day mortality and a significant difference in s-ICU/ICU admission [respectively, χ^2^(1) = 0.992, *p* = 0319; and χ^2^(1) = 23.62, *p <*0.001, **Figures 2A, B**].

A binomial logistic regression analysis was then performed to ascertain the effects of all the aforementioned variables, different from hypogammaglobulinemia, on the likelihood of admission to s-ICU/ICU in the whole cohort of patients hospitalized for COVID-19 pneumonia, as summarized in **Table 5**. Bronchiectasis (OR = 27.52, *p <*0.001), superinfections (OR = 4.18, *p <*0.001), cancer (OR = 3.05, *p <*0.001), haematological malignancies (OR = 4.69, *p <*0.001) and active cancer treatment (OR = 5.53, *p <*0.001), ischemic heart disease (OR = 2.27, *p* = 0.042), length of hospital stay (OR = 1.20, *p <*0.001), CRP (OR = 1.08, *p <*0.001) and PCT (OR = 1.24, *p* = 0.030) were the nine variables significantly associated with an increased likelihood of admission to s-ICU/ICU. Male gender and dyslipidemia were found close to statistical significance. Multivariable logistic regression confirmed the impact of hypogammaglobulinemia on the disease severity (Score ≥5, OR 8.52 95% CI 3.31–21.93 *p <*0.001, **Table 4**) and the probability of sICU/ICU admission (OR 5.35 95% CI 2.08–13.78 p <0.001, **Table 4**) after adjustment for sex, age, steroid treatment and presence of cancer. Adjustment for haematological malignancies instead of cancer in general did not disprove the impact of hypogammaglobulinemia (**Supplementary Material** and **Supplementary Figure 4**). Correspondingly, the Cox proportional hazard regression was then used to adjust survival analysis for the same selected confounders, confirming the impact of hypogammaglobulinemia on sICU/ICU admission (HR 3.73, *p <*0.001, **Supplementary Figure 3A**). Multivariable logistic regression and Cox proportional hazard regression confirmed the absence of significant correlation between hypogammaglobulinemia and mortality within 30 day since admission (**Table 4** and **Supplementary Figure 3B**).

**Table 5:** Binomial logistic regression between ICU *vs.* no-ICU in overall COVID-19 cohort.

	OR (95% CI)	p*
Age	1.00 (0.98–1.03)	0.789
Gender	1.89 (0.97–3.70)	0.062
Time to hospitalization	1.03 (0.95–1.11)	0.521
Cancer	3.05 (1.65–5.67)	**<0.001**
Haematological malignancies	4.69. (2.14–10.31)	**<0.001**
Active cancer treatment	5.53 (2.33–13.20)	**<0.001**
Arterial hypertension	0.94 (0.51–1.74)	0.835
Dyslipidemia	1.87 (0.98–3.55)	0.057
Diabetes mellitus	0.917 (0.44–1.93)	0.820
Ischemic heart disease	2.27 (1.04–4.96)	**0.040**
Obesity	0.43 (0.15–1.24)	0.116
Bronchiectasis	27.52 (7.26–104.30)	**<0.001**
WBCs (mm*^3^*)	1.06 (0.98–1.15)	0.136
Lymphocytes (mm*^3^*)	1.03 (0.99–1.08)	0.101
CRP (mg/dl)	1.08 (1.04–1.13)	**<0.001**
ERS (mm/h)	1.01 (0.99–1.03)	0.168
PCT (ng/ml)	1.24 (1.02–1.51)	**0.030**
IL-6 (mg/dl)	1.40 (0.87–2.28)	0.168
D-Dimer (ng/l)	1 (1–1)	0.470
Ferritin (ng/ml)	1 (1–1)	0.339
Superinfections	4.18 (2.05–8.52)	**<0.001**
Thromboembolism	0.31 (0.04–2.36)	0.258
Disease duration	1.01 (0.98–1.05)	0.514
Length of hospital stay	1.20 (1.15–1.26)	**<0.001**

*Binomial logistic regression analysis.

Bold font highlights statistically significant p values.

### sICU/ICU Versus Non-ICU in HYPO Cohort

Taking into consideration the admission to sICU/ICU among the HYPO population, the median age (75 vs. 65 mg/dl, *p* = 0.152) and the gender distribution expressed as male percentage of the total (60 vs. 37.5%, *p* = 0.170) were not different between patients admitted or not to sICU/ICU. **Supplementary Table 2** shows the distribution of comorbidities among sICU/ICU HYPO vs. no-sICU/ICU HYPO group, with a borderline significant difference only for bronchiectasis (40 vs. 12.5%, *p* = 0.047). Of note, we found no difference.

We found a significant difference in the use of IgRT during the hospitalization (0% sICU/ICU HYPO vs. 33.3% no-sICU/ICU HYPO, *p* = 0.012). Of note, none of the 8 HYPO patients who received IgRT was admitted to sICU/ICU or died. No differences were detected in the use of convalescent plasma, SARS-CoV-2 Monoclonal Antibodies, Remdesivir, antibiotics and steroid therapy (**Table 6**). As expected, there was a higher prevalence of mechanical ventilation in the sICU/ICU group (100 vs. 45.8%, *p <*0.001).

**Table 6:** Clinical and laboratory outcomes between ICU *vs.* no-ICU among the HYPO cohort.

*HYPO cohort*	*ICU (n = 15)*	*no-ICU (n = 24)*	p*
**Age**—years (IQR)	75 (61–82)	65 (58–79)	0.152
**Gender**—% Male	60%	37.5%	0.170
**Treatments**—n%			
*Remdesivir*	3 (20%)	7 (29.2%)	0.524
*Steroid therapy*	11 (73.3%)	17 (70.8%)	0.866
*Antibiotics*	12 (80%)	16 (66.7%)	0.368
*Mechanical Ventilation*	15 (100%)	11 (45.8%)	**<0.001**
*IgRT during hospitalization*	0 (0%)	8 (33.3%)	**0.012**
*SARS-CoV-2 Monoclonal Antibodies*	1 (6.7%)	1 (4.2%)	0.651
*Convalescent plasma*	1 (6.7%)	4 (16.7%)	0.363
**Laboratory**—unit (IQR)			
*IgG—mg/dl (IQR)*	488 (420–533)	468 (326–523)	0.596
*IgA—mg/dl (IQR)*	52 (48–70)	102.5 (60–117)	0.099
*IgM—mg/dl (IQR)*	32 (19–42)	17.5 (9–56)	0.635
*WBCs (mm)*	8.7 (6.9–10.2)	8.1 (6.7–10)	0.074
*Lymphocytes (mm^3^)*	1.1 (0.6–1.4)	0.66 (2.5–1)	0.483
*CRP (mg/dl)*	19.3 (9.5–33.5)	12.5 (2.5–16.7)	**0.003**
*PCT (ng/ml)*	2 (1–3)	0.05 (0–2)	**0.012**
*IL-6 (mg/dl)*	35.5 (20.3–47.6)	12.5 (9.6–17.8)	**0.001**
**Complications and disease duration**			
*Superinfections—n (%)*	8 (53.3%)	11 (45.8%)	0.648
*Thromboembolism*	1 (2.6%)	0 (0%)	0.423
*Disease duration—days (IQR)*	22 (20–40)	30 (20–35)	0.675
*Time to hospitalization—days (IQR)*	7 (4–11)	7 (4–8)	0.502
*Length of hospital stay—days (IQR)*	20 (16–28)	14 (11–21)	**0.007**
*Mortality—n (%)*	6 (40%)	3 (12.5%)	**0.047**

Quantitative variables are expressed as median and IQR. Categorical variables were reported as percentages of the total. The sex ratio is expressed as male percentage of the total. *Chi-squared and Mann–Whitney test.

HYPO, hypogammaglobulinemia; WBCs, total white blood cell; CRP, C-reactive protein; PCT, procalcitonin; IL-6, interleukin-6.

Bold font highlights statistically significant p values.

Considering the laboratory parameters (**Table 6**), we found that sICU/ICU HYPO group presented a significant increase of the inflammatory markers, respectively CRP (19.3 vs. 12.5 mg/dl, *p* = 0.003), PCT (2 vs. 0.05 mg/dl, *p* = 0.012) and IL-6 (35.5 vs. 12.5 mg/dl, *p* = 0.001). No differences were found for WBCs, lymphocytes count, serum IgA, IgM and IgG (488 vs. 468 mg/dl, *p* = 0.596).

**Table 6** also underlines that HYPO patients admitted to sICU/ICU presented significantly longer hospitalization (20 vs. 14 days, *p* = 0.007), but no significant differences in terms of time to viral clearance, time from the onset to admission, superinfections and thromboembolism. As expected, comparing the mortality rate between sICU/ICU and no-sICU/ICU HYPO patients we found a higher prevalence of deaths in sICU/ICU than no-sICU/ICU (40 vs. 12.5%, *p* = 0.047).

## Discussion

Our study specifically explores the impact of hypogammaglobulinemia, regardless of its cause, on the course of COVID-19 in consecutive patients hospitalized in a non-intensive care setting. Previous studies, differently, explored the course of the disease in well-defined cohorts of primary immunodeficiency or hematologic patients (11, 18–20). Studies on hematologic patients, in particular, included hospitalized and non-hospitalized subjects, mainly grouped for specific diseases and without focusing on hypogammaglobulinemia in the in-patient setting (18, 20). Moreover, the role of IgRT has not been investigated at all (11, 18–20).

In our whole cohort, we found age, sex and mortality similar to what resulting from the epidemiological metanalysis performed in our region (21). Despite a relatively low threshold adopted for gamma-globulin and IgG serum levels, we found more than 10% of admitted patients presenting antibody deficiency, of which only one had primary antibody deficiency. This prevalence is apparently high, but possibly related to age and comorbidities (22).

At least 25% of the patients with hypogammaglobulinemia received a new diagnosis of hypogammaglobulinemia during the hospitalization, despite previous clinical records demonstrating an un-recognized pre-existing antibody defect for all of them. Hypogammaglobulinemic patients presented a significantly increased frequency of malignancies (of hematologic malignancies, in particular), active anti-neoplastic treatment and bronchiectasis; all these variables are potentially related to the presence of hypogammaglobulinemia and to a severe COVID-19 course (12, 23, 24). No differences were detected between patients with and without hypogammaglobulinemia in terms of age, gender and cardiovascular co-morbidities. On the contrary, patients with hypogammaglobulinemia more frequently presented superinfections and underwent immunoglobulin-based treatments (convalescent plasma, monoclonal and polyclonal immunoglobulins), and also antibiotic treatment, while steroids were less used. This latter was an unexpected finding; we might speculate that hypogammaglobulinemia could have been considered a relative contraindication, for some patients, in steroids prescription. As already reported for PAD patients (11), our patients with hypogammaglobulinemia also presented longer disease duration and hospitalization. These might in part be due to an impairment in viral clearance and to the increased rate of superinfection (10). As a consequence, hypogammaglobulinemia was also associated to a more severe COVID-19 course, with higher need for non-invasive and invasive ventilation, higher score of disease severity and more frequent admission to s-ICU/ICU, despite all enrolled patients presenting a seven-category score ≤4 at admission. No difference in mortality has been instead detected between the two cohorts of patients, with a rate comparable to the national rate for the general population, suggesting that antibody deficiency itself does not impact on mortality, as already suggested for IEIs (11). However, this specific finding deserves further investigations on larger cohorts.

The correlation of hypogammaglobulinemia with disease severity and sICU/ICU admission was confirmed when adjusting our analysis for possible confounders, namely gender, age and presence of cancer. Since hematologic malignancies were differently represented between hypogammaglobulinemic patients and controls, we also specifically ruled out that their presence might have itself explained the difference in COVID-19 severity. When replacing cancer with haematological malignancies in the multivariate regression analyses and in the Cox proportional hazard regression for sICU/ICU admission, the significance of the model and the impact of hypogammaglobulinemia did not change. We did not include superinfections and bronchiectasis in our final model, due to their likelihood of dependence on hypogammaglobulinemia itself (25–27). For the same reason we also selected cancer instead of hematologic malignancies or active cancer treatment.

Thus, our study supports the hypothesis that, in hospitalized patients, a condition of antibody deficiency may represent a risk factor for a longer and more severe clinical course in SARS-CoV-2 infection, particularly in absence of immunoglobulin replacement therapy during hospitalization (only two patients were receiving regular IgRT before hospital admission).

The inter-relationship between cancer and its treatment, hypogammaglobulinemia, bronchiectasis, and superinfections needs to be taken into account, both in terms of causality and synergistic effect. Indeed their co-occurrence might reasonably enhance COVID-19 severity. Bronchiectasis had already been included in proposed models of disease severity prediction (28), and also oncologic and onco-hematologic diseases that might broaden the risk for infectious (including opportunistic) complications, as for the immunoparesis in MM (18–20, 29). Compared to the above mentioned, however, the advantage of hypogammaglobulinemia is the possibility of promptly restoring and keeping normal IgG serum levels by IgRT (30).

In our cohort, the extremely low rate of patients with antibody deficiency already undergoing IgRT at the time of admission raises at least two main considerations. The first one concerns the role of a regular IgRT in preventing a more severe course of COVID-19, considering data coming from PAD patients regularly receiving IgRT (11). The second one is the age-old question of underdiagnosis and undertreatment of hypogammaglobulinemia (31, 32). Current evidence on IgRT administration during COVID-19 suggest to continue the routine administration of replacement therapy during the infection and, in case of hospitalization, a prompt evaluation of IgG trough level is required to assess and boost up the dosage (33, 34).

A further question is whether or not a prompt administration of intravenous immunoglobulin (IVIg) in those patients found with hypogammaglobulinemia when admitted for COVID-19 might somehow impact on the disease course. Our study seems to support such a view, since the 8 patients with hypogammaglobulinemia who underwent, for the first time, a single intravenous immunoglobulin (IVIg) administration at standard replacement dosage during hospitalization completely recovered without requiring s-ICU/ICU admission. Accordingly, even in the case of SARS and Middle East respiratory syndrome IVIg therapy provided clinical benefits (35). We may also consider that IVIg may contain cross-reactive antibodies against SARS-CoV-2 due to common circulating coronaviruses (36). Moreover, recent evidence suggests that the content of anti-SARS-CoV-2 neutralizing antibodies in commercially available IVIg preparation is increasing over time, thanks to healed and vaccinated donors (37).

Our sample is too small to draw any firm conclusion. However, it strengthens the need for exploring the possible systematic application of at least extemporary IgG replacement in patients with hypogammaglobulinemia at admission. In this view neutralizing antibodies, unlikely to be present in the Ig preparation at the end of 2020/beginning of 2021, might now further enhance the impact of IVIg. We might at least hypothesize that an extemporary administration of IgRT could help to prevent bacterial superinfections that might in turn contribute to the inflammatory burden and to the need for ventilatory support. IgRT could also be easily combined with monoclonal antibodies administration, since no relevant interaction is expected (38, 39).

Finally, immunomodulatory effects of IVIg both on innate and adaptive immunity might also play a role in the viral infection and may represent a useful therapeutic tool in the early stages of SARS-CoV-2 infection (40, 41). Evidence of successful usage of IVIg at a dose of 0.3–0.5 g/kg continuously for five consecutive days, in addition to the standard of care, has been reported and seems to reduce the need for mechanical ventilation and to improve the disease course (42–44).

This study has many limitations, starting from the relatively small size of the cohort and the retrospective design. Moreover, the HYPO group is constituted by patients with different conditions underlying hypogammaglobulinemia and by others with hypogammaglobulinemia of undefined significance/origin. This does not allow drawing specific conclusions about the impact of different types of hypogammaglobulinemia on COVID-19 course. Finally, numbers are too small to gain definite information on the impact of IgRT. Despite that, the adjustment for concomitant treatments, cancer and, more specifically, haematological conditions did not impact on the relation between hypogammaglobulinemia and a worse COVID-19 course in admitted patients and IgRT displayed an apparently favorable impact on disease course. All these considered, our study findings emphasize the add-value of routine serum protein electrophoresis evaluation in patients admitted with COVID-19 to support clinicians in patient care and to consider initiation of IgRT during the hospitalization. The accessibility of the test and the availability of the treatment deserve further prospective investigations.

## Data Availability Statement

The raw data supporting the conclusions of this article will be made available by the authors, without undue reservation.

## Ethics Statement

The studies involving human participants were reviewed and approved by the Comitato Etico per la Sperimentazione Clinica delle Province di Treviso e Belluno (Study number 793/CE Marca). The patients/participants provided their written informed consent to participate in this study.

## Author Contributions

AD, FC, RS, and MR designed the study. FC coordinated the study and the writing. FC, RS, and AD performed the statistical analysis and wrote the paper. RS and AD equally contributed. ST, FM, AD, and RFG collected data from clinical records. RB, FC, CFa, and MG were involved in the clinical management of COVID-19 patients. GB performed the laboratory analysis and collected lab data. All authors contributed to the critical revision of the manuscript. MR finally revised the paper. All authors listed have made a substantial, direct, and intellectual contribution to the work and approved it for publication.

## Funding

Funding for open access publication will be partially granted by the Fondazione Cariparo, through the Department of Medicine of the University of Padova. Fondazione Cariparo CoviDimed project (protocol n. 55813).

## Conflict of Interest

The authors declare that the research was conducted in the absence of any commercial or financial relationships that could be construed as a potential conflict of interest.

## Publisher’s Note

All claims expressed in this article are solely those of the authors and do not necessarily represent those of their affiliated organizations, or those of the publisher, the editors and the reviewers. Any product that may be evaluated in this article, or claim that may be made by its manufacturer, is not guaranteed or endorsed by the publisher.
