# Supplementary material for: Impact of Hypogammaglobulinemia on the Course of COVID-19 in a Non-Intensive Care Setting: A Single-Center Retrospective Cohort Study
Source: Front Immunol. 2022 Mar 10;13:842643. doi: 10.3389/fimmu.2022.842643 (PMC8960988; doi:10.3389/fimmu.2022.842643)
Supplement: Supplementary file 1 [file DataSheet_1.pdf]

SUPPLEMENTARY MATERIAL

**Microbiological evidences**	**HYPO** (n=39)	**no-HYPO** (n=335)
Staphylococcus aureus	5	3
Streptococcus pneumoniae	5	3
Enterococcus faecium	2	2
Escherichia coli	2	2
Pseudomonas aeruginosa	2	1
Cytomegalovirus	3	0
Aspergillus fumigatus	2	0
Clostridium difficile	0	1
Klebsiella pneumoniae	3	1

Supplementary Table 1.
Different pathogens in microbiological samples (blood cultures, urine cultures and swabs) between HYPO and no-HYPO groups in our COVID-19 cohort.
HYPO = hypogammaglobulinemia.

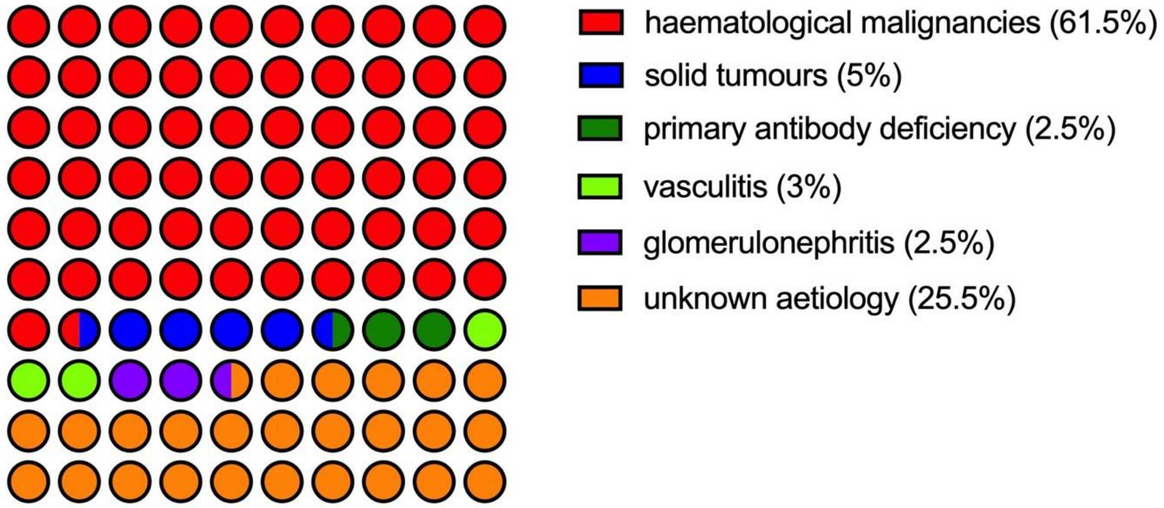

Supplementary figure 1. Different aetiologies of hypogammaglobulinemia in our COVID-19 cohort are expressed as percentage of the total (HYPO group, n = 39 cases).

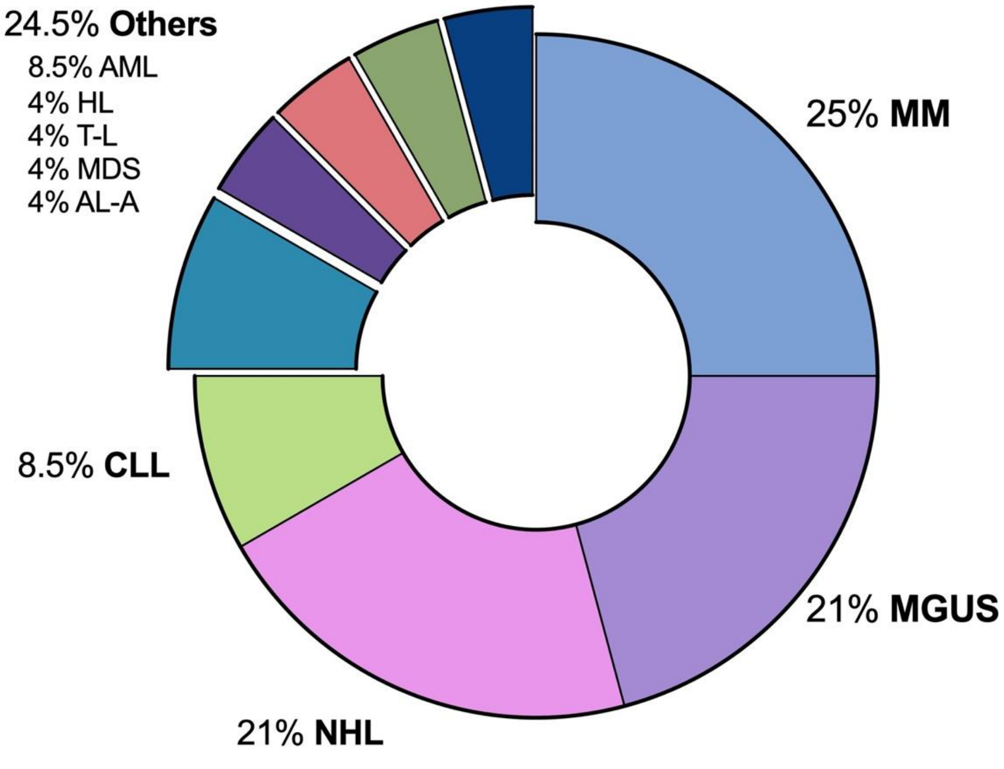

Supplementary Figure 2. Different causes of hypogammaglobulinemia in the haematological subgroup of hypogammaglobulinemia (n = 24, 61.5% of HYPO group).

MGUS = monoclonal gammopathy of undetermined significance, MM = multiple myeloma, NHL = non-Hodgkin's lymphoma, CLL = chronic lymphocytic leukaemia, AML = acute myeloid leukaemia, HL = Hodgkin's lymphoma, T-L = T-cell lymphoma, MDS = myelodysplastic syndrome, AL-A = AL amyloidosis.

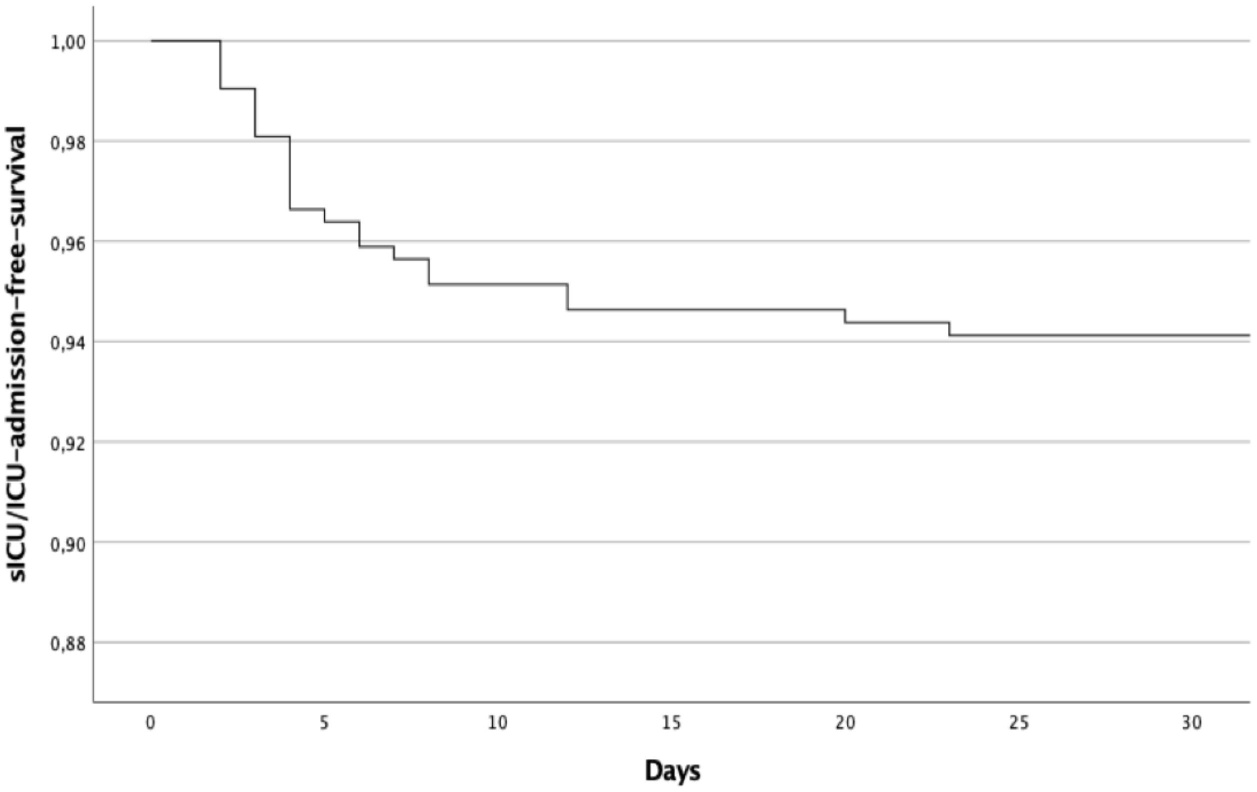

Supplementary Figure 3 A. Survival analysis of sICU/ICU admission within 30 day adjusted for HYPO, age, sex, cancer and steroid therapy. Days are calculated since the admission to the hospital. Cox Regression HR adjusted: 3.73 (1.75-7.91), $p < 0.001$.

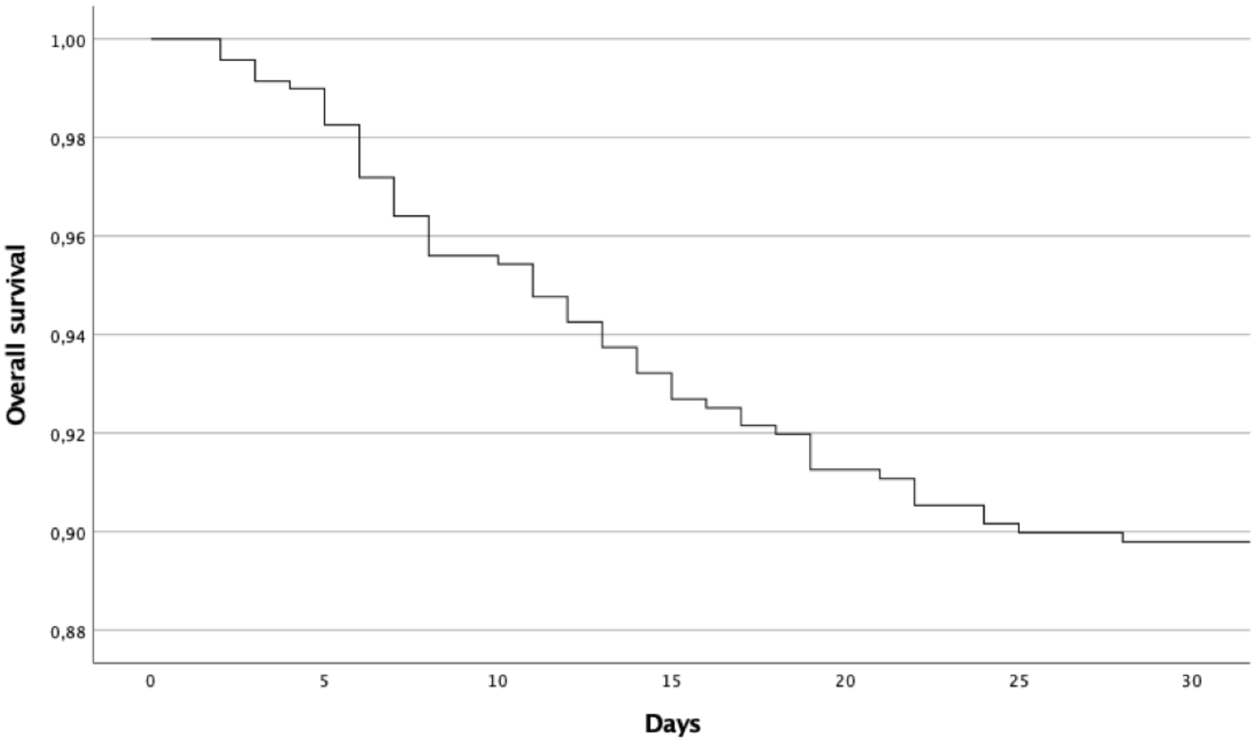

Supplementary Figure 3 B. Survival analysis of mortality within 30 day since admission adjusted for HYPO, age, sex and cancer and steroid therapy. Days are calculated since the admission to the hospital. . Cox Regression HR adjusted: 1.42 (0.62-3.23), *p* = 0.404.

Haematological malignancies were differently represented between HYPO and non-HYPO patients, as evidenced by the binomial regression analysis (p<0.001, OR 4.69. CI95% 2.14-10.31I). However, when replacing cancer with hematological malignancies in the multivariate regression analyses, the significance of the model and the impact of hypogammaglobulinemia did not change. When considering the outcome "ICU admission" we found p=0.002 for hypogammaglobulinemia, with OR 4.75 CI95% 1.80-12-52, whereas p=0.166 for hematological malignancies, with OR 2.08 CI95% 0.74-5.85. In case of Severity score >=5 we found p<0.001 for hypogammaglobulinemia, with OR 12.78 CI95% 4.52-36.14, whereas p=0.784 for hematological malignancies, with OR 0.86 CI95% 0.29-2.54. As a second control, we adjusted for hematological malignancies, age, sex and hypogammaglobulinemia the Cox proportional hazard regression for sICU/ICU admission, again finding a consistency of hypogammaglobulinemia with a HR of 2.86 (1.15-7.13), p = 0.024 (see Supplementary Figure 4 below).

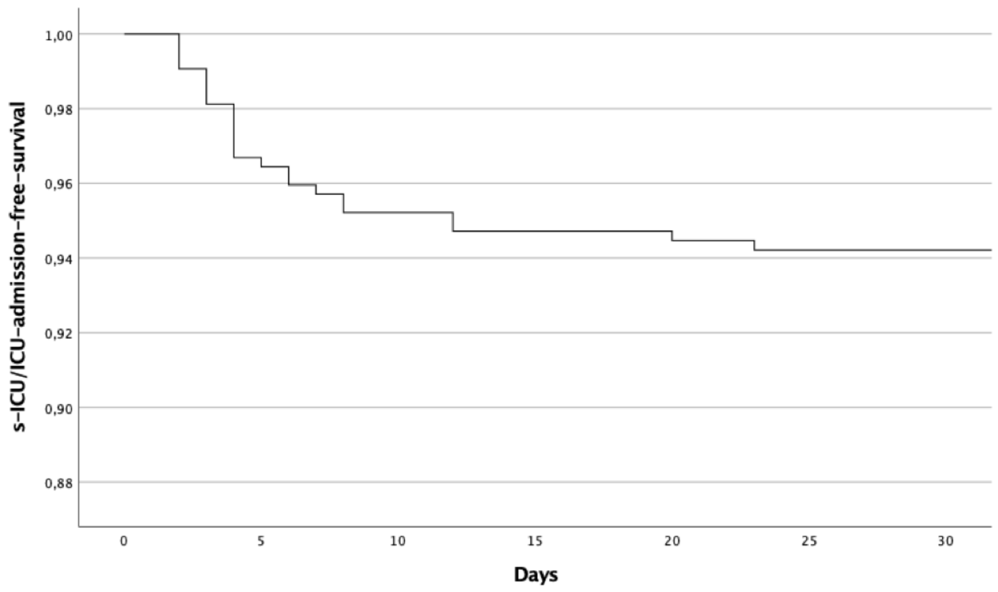

Supplementary Figure 4. Adjust survival analysis of sICU/ICU admission within 30 day for HYPO, age, sex and hematological malignancies. Days are calculated since the admission to the hospital. Cox Regression HR: 2.86 (1.15-7.13), *p* = 0.024.

	**ICU** (n=15)	**no-ICU** (n=24)	***p****
**Comorbidities n (%)**			
Cancer	10 (66.7%)	16 (66.7%)	1
Active cancer treatment	7 (70%)	11 (68.8%)	0.946
Bronchiectasis	6 (40%)	3 (12.5%)	**0.047**
Arterial hypertension	8 (53.3%)	11 (45.8%)	0.648
Dyslipidemia	5 (33.3%)	7 (29.2%)	0.784
Diabetes mellitus	2 (13.3%)	4 (16.7%)	0.779
Ischemic heart disease	2 (13.3%)	5 (20.8%)	0.553
Obesity	1 (6.7%)	4 (16.7%)	0.363
Previous corticosteroid therapy	8 (53.3%)	8 (33.3%)	0.217

Supplementary Table 2.
Comparison of comorbidities between ICU *vs.* no-ICU among the HYPO cohort.
*Chi-squared test.
